# Modified Transepithelial Phototherapeutic Keratectomy for Band Keratopathy †

**DOI:** 10.3390/jcm13195717

**Published:** 2024-09-25

**Authors:** Rachana Prashant Shah, Mayank A. Nanavaty

**Affiliations:** 1 Sussex Eye Hospital, University Hospitals Sussex NHS Foundation Trust, Eastern Road, Brighton BN2 5BF, UK; 2 Brighton & Sussex Medical School, University of Sussex, Falmer, Brighton BN2 5BF, UK

**Keywords:** band keratopathy, excimer laser, chelation

## Abstract

**Objectives:** To report the outcomes of novel modified transepithelial phototherapeutic keratectomy (PTK) in treating band keratopathy (BK). **Methods:** A retrospective analysis was performed on patients who underwent PTK for BK at the Sussex Eye Laser Clinic, Nuffield Health, Brighton. Patients with BK obscuring the visual axis, affecting visual acuity, or causing discomfort were considered for PTK. All the patients underwent preoperative evaluation, including preoperative corneal topography and optical coherence tomography. Modified transepithelial PTK was performed without using EDTA for chelation or alcohol for epithelium debridement. Patients were followed up for one week and then every two weeks after that until two months. Preoperative and postoperative best corrected visual acuities (BCVA) were compared using a paired *t*-test. **Results:** We studied 15 eyes of nine patients undergoing novel PTK for BK. The mean age was 80 ± 5.73 years. The mean pre-treatment visual acuity was 0.68 ± 0.17 logMAR (range: 0.6 logMAR to 1 logMAR) and improved to 0.22 ± 0.09 logMAR (*p* < 0.05), ranging from 0.18 to 0.48 logMAR at two months following PTK. None of the patients complained of ocular discomfort following the procedure. A repeat procedure was not required for any of these patients. **Conclusions:** Modified transepithelial PTK is an effective procedure for improving visual outcomes in patients with band keratopathy and should be considered for the treatment of band keratopathy.

## 1. Introduction

Band keratopathy (BK) is a superficial corneal degeneration consisting of band-shaped fine calcium deposits in the sub-epithelium, Bowman’s layer and the anterior stroma of the interpalpebral cornea [1,2]. In eyes with a rough calcified surface, there was often severe underlying pathology, such as penetrating trauma, previous herpetic disease, severe uveitis, and end-stage glaucoma [3]. In patients with smooth bands often no predisposing cause may be found. Band keratopathy has been reported following long term pilocarpine treatment [4] and is thought to result from the presence of phenylmercuric nitrate preservatives. It is also known to occur in patients taking vitamin D supplements and disorders of calcium metabolism [5,6]. Histologically, there may be calcific or non-calcific deposits [1,7].

Although early stages are asymptomatic, the accumulation of deposits can cause significant ocular discomfort, irritation, photophobia and recurrent corneal erosions. Deposits obscuring the visual axis can lead to visual disturbances. Also, severe BK affects the diagnosis and treatment of other concomitant intraocular diseases [8]. The surgical approach for BK is indicated after treating any other concomitant systemic or ocular pathologies. The surgical techniques mainly aim at removing these opaque deposits and have been previously reported as below:

1. Superficial Keratectomy/Mechanical debridement: The main advantages associated with this technique involve the ease of surgery and minimal cost involved. In cases where the calcium plaque is thick, it can be scraped off the cornea with forceps; alternatively, a superficial keratectomy with a blade can be performed. However, it can lead to irregular surface post debridement as the depth of the treated area is not predictable [9].
2. EDTA chelation [10,11,12,13,14]: By far the most widely used method is ethylenediamine- tetraacetic acid (EDTA) chelation which helps in sequestering the calcium deposits. This technique can be used in isolation or supplemented with manual superficial keratectomy. The main advantage of EDTA chelation over mechanical debridement alone is that the EDTA only removes the calcium sparing the normal tissue. The procedure involves the application of 0.05 mol/l EDTA to the subepithelial calcification with a surgical sponge or directly using a reservoir. The chemical reaction takes around 5–30 min depending upon the density of calcium deposits. Then, the loosened calcium deposits can be removed using blunt dissection with cellulose sponges or gentle scraping. A technique using alcohol to elevate and preserve the epithelium, use of EDTA and then replacement of the epithelial sheet similar to LASEK to provide better analgesia has also been described [15]. The drawback of EDTA is that it does not act on non-calcific deposits.
3. Diamond burr [16]: Using the battery-powered 5.0 mm diamond-dusted burr, gentle and even pressure is applied to the cornea in either a rotary or a back-and-forth manner until the calcium is removed.
4. Amniotic membrane transplantation (AMT) [17,18,19,20]: The amniotic membrane provides a mechanical bandage that protects corneal wounds, absorbs inflammatory cytokines, and reduces pain during corneal wound healing. The rationale behind using AMT in the treatment of BK is that it facilitates healing and provides long-term stability to the corneal epithelium. AMT can be used as an adjunct to augment conventional surgical and chemical removal of BK by replacing damaged the basement membrane. Protease inhibitors in the amniotic membrane can inhibit potential surface neovascularization.
5. Nd YAG laser [8]: The Nd:YAG laser acts only on the calcium deposits and the energy only reaches the level of the deposits, without affecting the deeper layers of the cornea. The controlled and uniform depth of total disruption results in smoother healing of the epithelium and an even corneal surface after the treatment.
6. Excimer laser phototherapeutic keratectomy (PTK) [2,3,7,21]: Since the late 1980s, the excimer argon fluoride (ArF) laser has been used to reshape the anterior corneal curvature in a procedure known as photorefractive keratectomy or keratomileusis in situ. The excimer laser emits pulses of ultraviolet light that ablate the cornea with submicron precision and minimal distortion to adjacent tissue [22].

The surgical approach for BK is indicated after treating any other concomitant systemic or ocular pathologies. Since the late 1980s, the excimer laser has been used to reshape the anterior corneal curvature in a procedure known as photorefractive keratectomy or keratomileusis in situ. Excimer laser PTK is an effective treatment for corneal surface irregularity, epithelial instability, and superficial opacification [7,23,24]. Non-calcific material deposition may account for the poor response to chelating agents such as ethylene diamine tetra-acetate (EDTA), as seen in some eyes. Hence, there is a need to resort to surgical management [3].

Surgical treatment aims to remove the opaque calcium deposits and/or restore the normal smooth corneal surface [3]. The advantage of lasers over manual superficial keratectomy is their ability to remove the corneal tissue with extreme precision and minimal adjacent tissue damage. Secondly, its large beam cross sections allow simultaneous treatment of wide areas [25]. This study was performed to determine the outcomes of patients who underwent our modified excimer laser ‘non-touch’ transepithelial PTK technique for BK.

### Patients and Methods

A retrospective analysis was performed on patients who underwent PTK for BK between June 2017 and May 2024 at the Sussex Eye Laser Clinic (https://www.sussexeyelaserclinic.co.uk/, accessed on 15 August 2024), Nuffield Health, Brighton, UK. Informed consent was obtained from the patient, and our study adhered to the tenets of the Declaration of Helsinki.

**Figure 1:**
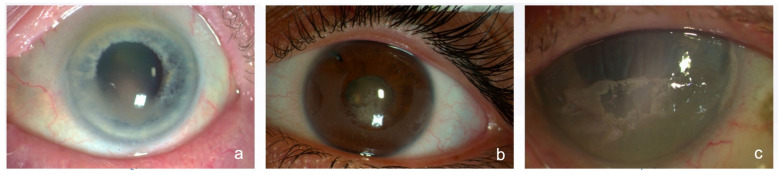
(**a**) Case of mild band keratopathy which can be suitable for ‘no touch’ transepithelial PTK. (**b**) Case of moderate band keratopathy which can be suitable for ‘no touch’ transepithelial PTK. (**c**) Case of thick band keratopathy with corneal oedema which is not suitable for ‘no touch’ transepithelial PTK.

Patients with BK obscuring the visual axis, affecting visual acuity (Figure 1a,b) or causing discomfort were considered for PTK after thoroughly discussing the treatment options with them. Those with associated corneal oedema (Figure 1c) or deep and extensive corneal scarring were excluded from the study. Patients with systemic diseases affecting the healing process, such as uncontrolled diabetes or collagen vascular diseases, were excluded.

All the patients underwent a thorough slit lamp examination. Preoperative corneal topography was performed to study preoperative refractive error, which could also be treated with PTK in cases of very mild BK. Optical coherence tomography (Solix, Visionix, Pont-de-l’Arche, France), both preoperative and postoperative, was performed to assess the depth of the cornea and ensure that at least 250 microns of residual stroma remained after the laser treatment [26]. Epithelial mapping was also performed on all cases however as the data on epithelial mapping are unreliable in irregular band keratopathy, these data were not included in the analysis of this study.

Wavelight EX500 193 nm excimer laser (Alcon Laboratories, Fort Worth, TX, USA) was used for performing PTK to restore a smooth corneal surface and reduce opacification caused by calcium deposits. Prior to the surgery topical anaesthetic (g. proxymetacaine 0.5% minims, Bausch & Lomb, Bridgewater, NJ, USA) was instilled, and antiseptic was achieved using g. povidone iodine 5%. Patients were asked to fixate, and fixation was assessed using an iris tracker intraoperatively. When the opacity of the BK was dense, iris tracking was disabled manually. Patients were also warned about the noise associated with the procedure. First, PTK was performed with a central 9.9 mm ablation and 8.0 mm optical zone, centred on the visual axis and with a depth of 40 microns. The second PTK was performed with a depth of 20 or 30 microns, depending on the remaining calcium deposits and the optical and ablation zones were titrated as per the residual BK. A further 20–30 micron depth was applied based on residual calcium deposits/opacification. Adequate ablative depth and residual stromal bed were assessed by regular visualization of the cornea between laser bursts down the operating microscope and the slit lamp on the laser. Further small-depth PTK ablations were performed until no deposits were noted under the operating microscope and slit lamp. Sodium hyaluronate 0.2% (HyloForte, URSAPharma, Saarbrücken, Germany) was used as a masking agent as and when needed. Mitomycin C 0.25 mg/0.5 mL drops were instilled and kept for 20 s in all cases at the end of the procedure before washing it out thoroughly with a balanced salt solution. A bandage contact lens was inserted. At the end of the procedure, the corneal surface was washed out and a bandage contact lens (BCL) was inserted. Patients were prescribed Ofloxacin 0.3% (EXOCIN^®^ 3 mg/mL) and prednisolone acetate 1% (Pred Forte, Allergan, Dublin, Ireland) four times a day for 14 days along with G. Proxymetacaine Hydrochloride 0.5% (Bausch & Lomb Minims, Bridgewater, NJ, USA) minims to be used up to 8 times a day for first 3 days only. The patients were instructed to use the Proxymetacaine Hydrochloride 0.5% as less frequently as possible but not to exceed up to 8 times a day for up to the first 3 days only. Only one box of Proxymetacaine Hydrochloride 0.5% containing only 20 minims were given to the patients. The patients were seen between a week and two following the procedure, BCL was removed at this stage and any pain beyond 3 days postoperatively was documented in the notes.

Patients were followed up in the clinic at one week and every two weeks thereafter for two months. Refraction was performed at the 2nd monthly visit, and any refractive error was treated with glasses, soft contact lenses, rigid contact lenses, or scleral contact lenses. An Excel spreadsheet (Microsoft, Corp., Redmond, WA, USA) was used for data analysis, from which the mean and standard deviation were calculated for continuous data and as a number with a percentage for categorical data. Pre-operative and post-operative best corrected visual acuities were compared using a paired *t*-test. A *p*-value less than 0.05 was considered statistically significant.

## 2. Results

We studied 15 eyes of nine patients undergoing our ‘no-touch’ transepithelial PTK for BK (Table 1). All the patients were treated by a single surgeon (MAN). The mean age was 80 ± 5.73 years, ranging from 72 to 90 years. In total, 6 out of 15 (40%) eyes had posterior segment pathology and underwent PTK to facilitate posterior segment visualization. The mean pre-treatment visual acuity was 0.68 ± 0.17 logMAR (range: 0.6 logMAR to 1 logMAR) and improved to 0.22 ± 0.09 logMAR (*p* < 0.05), ranging from 0.18 to 0.48 logMAR at two months following PTK (Table 1). Three (20%) eyes had undergone previous EDTA chelation, which did not relieve the symptoms and underwent PTK. Out of seven patients with residual refractive error, one was prescribed a rigid gas-permeable contact lens in one eye and a soft contact lens in the other. The rest were prescribed glasses. None of the patients complained of ocular discomfort following the procedure with our postoperative medication regime. All the patients had fully healed epithelium at one week and one had any haze at 2 months. A repeat procedure was not required for any of these patients.

Following are some representative examples of patients who underwent our modified ‘no-touch’ transepithelial PTK technique for a case of mild BK and a case of thick BK:

1. The first case was a patient with the left eye having mild BK and a central corneal thickness of 498 microns. The first part of the treatment was performed with an optical zone of 8 mm and an ablation zone of 9.9 mm with a maximum depth of 40 microns. After assessing the amount of calcium deposits, the second part of the treatment was performed with a depth of 20 microns. Residual calcium deposits were treated with a maximum depth of up to 7.8 microns. The residual stromal bed following the procedure was more than 250 microns.
2. The second case was a patient with thick BK that required 1% hydroxyethylcellulose to mask the declivities between surface irregularities. The first part of the laser was applied with a maximum depth of 30 microns, and the second part with a depth of 20 microns. The residual deposits were treated with a third round of laser with a depth of 30 microns, followed by 20 microns. The cornea was examined for residual calcium deposits after each round of laser.

## 3. Discussion

Band keratopathy can lead to decreased visual acuity if it obscures the visual axis and ocular discomfort if it breaks the corneal epithelium, typically seen in rough bands. The main aim of treatment is to eliminate the corneal opacification caused by the deposits and to restore a smooth corneal surface. Various treatment options are available, including mechanical debridement, superficial keratectomy, EDTA chelation, and PTK [7,11,18].

Manual superficial keratectomy with EDTA chelation is a commonly practised treatment across the United Kingdom. However, manual superficial keratectomy lacks precision, and EDTA does not act on non-calcific deposits. In our experience, PTK provides better outcomes than conventional manual superficial keratectomy with EDTA. A study performed by Al-Hity and colleagues showed a recurrence rate of 28.1% following EDTA chelation [10]. This could be due to the non-calcific deposits not completely treated with EDTA. PTK can act on non-calcific deposits, is more precise, and restores a smooth corneal surface [3]. A study using an excimer laser for BK by O’Brart et al. showed an 88% improvement in glare and an 88% improvement in visual acuity [3]. Sharma et al. performed excimer PTK on post-silicone oil-induced BK in 20 eyes, with 90% showing an improvement in BCVA of two or more lines at six weeks [27]. Najjar et al. [11] reported the results of EDTA chelation in 54 cases with calcific band keratopathy. Treatment was performed using EDTA for 5–45 min depending upon the desired end-point of corneal clarity [11]. Nearly all (98%) patients reported symptomatic relief after the treatment. Recurrences were reported in 17.8% of cases. Recurrences following EDTA could be due to the non-calcific deposits not completely treated with EDTA. PTK can act on non-calcific deposits, is more precise, and restores a smooth corneal surface. Favourable ocular surface changes occur after PTK, including increased tear film stability owing to attaining a regular corneal surface and possibly through better mucin production by a healthier epithelium [28]. Heida and colleagues [29] conducted a study to investigate the clinical outcomes and recurrence of PTK for anterior corneal pathologies and divided it into six groups—granular dystrophy, BK, epithelium attachment disorder, gelatinous-drop-like corneal dystrophy, lattice dystrophy, and others. Significant improvement in corrected distance visual acuity was seen with a recurrence of BK in 4 out of 238 patients [29].

In a study performed by Bee et al. [16], a diamond-dusted burr was used to remove the calcium after debriding the epithelium on seven eyes of six patients with no visual. No chelating agent was used. They observed that two eyes experienced recurrence at 4 months and 28 months after the procedure. This is a good alternative for thick, raised corneal deposits with no visual potential but requires further large sample studies. However, in eyes with good visual potential, PTK is a better alternative considering the risk of infectious keratitis and stromal thinning associated with the use of diamond burr [30]. Baltatzis et al. [8] utilized Nd YAG laser for the treatment of BK in seven cases. They found complete removal of calcium deposits at the end of the procedure in six patients with no recurrences. Nd:YAG could be a satisfactory alternative; however, studies with a larger sample size and long-term follow-up are required.

Conventional PTK involves alcohol-assisted debridement of the epithelium followed by the application of a laser. Excimer lasers can remove thick and/or deep calcific plaques and non-calcific deposits, which other methods, such as EDTA chelation, may not achieve [7,15,31]. PTK can provide a smooth corneal surface for these patients after treatment [3,32]. As a variation in the PTK technique, transepithelial PTK has been used to treat various corneal pathologies, including superficial corneal opacification and irregular astigmatism [33]. Generally, alcohol-assisted epithelial debridement must be performed first for conventional PTK. Unlike conventional methods, transepithelial PTK uses a laser to remove the corneal epithelium directly, thus providing several advantages [33]. This procedure does not require intraoperative configuration and application of alcohol or other solutions, nor does it rely on masking agents used in areas of significant epithelial variability with corneal opacity [34]. As a result, it provides a shorter operation time and better intraoperative comfort than the conventional PTK technique. Epithelial removal using an excimer laser could provide a more uniform epithelial ablation compared with conventional methods thus resulting in faster epithelial healing with less pain and haze [35]. Naderi et al. [36] and Gaeckle [37] compared single-step transepithelial photo-refractive keratectomy (TE-PRK) and mechanical PRK and concluded that single-step TE-PRK offered faster visual recovery, epithelial healing and less pain compared with manual PRK. Similarly, Aslanides et al. [38] reported that single-step TE-PRK provided faster epithelial healing, lower pain scores, and significantly less haze formation compared with alcohol-assisted PRK. We performed a ‘no-touch’ technique of transepithelial PTK without using alcohol or EDTA. This eliminates the toxic effects of alcohol [35], increases intraoperative comfort, and allows faster epithelial healing as seen in our cohort. To our knowledge, this is one of the few studies on transepithelial PTK to treat band keratopathy. The disruption to the integrity of the epithelium and anterior stroma from the conventional technique involving removal of epithelium causes keratocyte migration, and the deposition of glycosaminoglycans and collagen into the anterior stroma during the healing phase [39]. If this causes clinically significant opacification, it is known as corneal haze; this can reduce the quality of vision significantly. Topical off-label mitomycin-C (MMC) is commonly used at the end of treatment to reduce haze through its mechanism of myofibroblast inhibition and reduction in keratocyte activity [40]. The advantage of our technique is that it can be customised to the thickness and extent of the BK. One can perform multiple rounds of variable thickness of PTKs and keep assessing the cornea for complete removal of calcium. In a study by Qian et al. [41], they treated 17 eyes with band keratopathy using transepithelial PTK. All their patients showed significant improvement in their symptoms and BCVA, with no recurrences during the follow-up period (3 to 19 months). They also stated that it was difficult to measure refractive status preoperatively, so optimal ablation depths and patterns of PTK treatment could not be determined [41]. This may result in unpredictable refractive changes postoperatively. The technique Qian et al. [41] used involved administering topical anaesthesia eye drops followed by transepithelial PTK using an excimer laser (AMARIS 500E, Schwind, Germany). They initially set the ablation depth at 150 microns with a residual stromal thickness of more than 350 microns after transepithelial PTK treatment. The actual ablative depth was reached once the cornea and iris details were deemed relatively clear by observation of the operating microscope. In milder cases of BK, preoperative corneal topography and OCT could be performed in milder cases of BK to determine the ablation depth and simultaneously treat refractive errors associated with mild BK.

Our technique has a unique advantage over the one described by Qian et al. In cases of very thick BK, both anterior segment optical coherence tomography and confocal microscopy performed preoperatively may not obtain accurate results [34,42]. Similarly, for the same reason, it is also challenging to measure refractive status preoperatively, which can result in unpredictable refractive changes postoperatively [43,44] which can be a disadvantage in Qian et al.’s technique where a single ablation depth of 150 microns is planned. However, with our modified technique, planning of smaller ablation treatment means that the ablations can be performed at small intervals to allow for adequate inspection of the cornea and the ablation can be stopped immediately (preventing further unnecessary ablation) once the calcific and non-calcific deposits have cleared on assessing through the surgical microscope and slit lamp.

Postoperative corneal pain is an issue with transPTK for BK. Various intra- and postoperative factors can help control postoperative pain. Certainly, the reduced thermal load offered by modern treatment platforms leads to reduced pain [45]. Our ‘no touch’ technique with staged small ablations helps reduce heavy single long thermal exposure. The many postoperative regimens available include topical NSAID drugs, topical steroids, cycloplegics, and oral analgesics [46,47]. Postoperative oral gabapentin was found to be ineffective in controlling postoperative pain [48]. A topical bandage contact lens soaked in ketorolac 0.45% reduced postoperative pain more than a bandage contact lens alone after transPTK [49]. Topical anaesthetics have been well employed in limited dilute concentrations to avoid epithelial toxicity [49]. The authors are aware of high-dose oral steroids being used in the perioperative period in conventional PTK and transPTK with proponents claiming a faster visual recovery, less pain, and minimal side effects if the steroids are tapered quickly.

Vitamin C levels in the tear film reduce after excimer laser ablations [49]. However, in a study by Alishiri et al. [50] oral supplementation did not improve haze, subjective pain, or re-epithelialisation time compared with a placebo in a study. Vitamin E is an antioxidant that may reduce keratocyte apoptosis after excimer laser ablation [51]. When combined together, vitamin A and E supplementation was associated with faster re-epithelialisation times and a slightly lower incidence of post-surface ablation haze [52]. Whether there is a true benefit to be found remains to be seen and further investigation is welcome in this area. However, in our study and clinical practice we do offer 1 g of vitamin C three times a day for the first 3 days.

The limitation of our study is that it is a retrospective study with a small sample size and some patients had both eyes included. Although no recurrences were found during the follow-up period in our study, a longer follow-up is required, considering the mean recurrence time reported to be 12–18 months [11]. Our technique aims to achieve a smooth corneal surface in the visual axis. If the patient has thick plaques on the limbus too, causing foreign body sensation, then it may need enlargement of laser ablation zones, slight decentration of zones or even a small amount of manual scraping of the plaques from the limbus. Comparative analysis of pre and postoperative tomo and topographic assessment is difficult in BK cases due to the variability of thickness of BK plaques. More prospective controlled trials are needed to study the long-term efficacy and recurrence rates of transepithelial PTK compared to other treatment options.

In summary, our novel no-touch trans-epithelial PTK technique is an effective procedure for improving visual outcomes with lower recurrence rates in the short term in patients with band keratopathy. Therapeutic excimer treatments are restricted to only certain parts of the UK. Our study suggests that PTK should be considered for the treatment of band keratopathy.

## Figures and Tables

**Table 1:** Patient demographics, indications, pre and postoperative visual acuity and means of optical correction postoperatively.

No. of Eyes	Age (Years)	Eye	Indication	Preoperative BCVA	Postoperative BCVA	Means of Correction Postoperatively
1	90	Right	Idiopathic	0.78	0.18	glasses
2		Left	Idiopathic	0.60	0.18	glasses
3	77	Right	Vit D suppl	0.60	0.18	glasses
4		Left	Vit D suppl	0.60	0.18	glasses
5	82	Right	Vit D suppl	0.48	0.18	unaided
6		Left	Vit D suppl	0.48	0.18	unaided
7	74	Right	ERM + VR surgery + prior failed chelation	1	0.18	RGPCL
8		Left	ERM + VR surgery + prior failed chelation	0.78	0.18	Soft CL
9	72	Left	Bilataral LASIK, idiopathic, prior failed chelation	0.60	0.18	unaided
10	84	Right	ERM + dry AMD	0.78	0.30	Glasses
11		Left	ERM + dry AMD	1	0.48	Glasses
12	85	Left	Vit D suppl	0.78	0.18	Glasses
13	77	Left	Vit D suppl + HSK	0.48	0.18	Glasses
14	80	Right	Vitelliform maculopathy	0.60	0.30	Glasses
15		Left	Vitelliform maculopathy	0.60	0.30	Glasses

BCVA = best corrected visual acuity; ERM = epiretinal membrane; AMD = age-related macular degeneration; HSK = herpes simplex keratitis; RGPCL = rigid gas permeable contact lenses; CL = contact lenses.

## Data Availability

The data presented in this study are available on request from the corresponding author.
